# Suid alphaherpesvirus 1 of wild boar origin as a recent source of Aujeszky’s disease in carnivores in Germany

**DOI:** 10.1186/s12985-023-02074-3

**Published:** 2023-06-01

**Authors:** Conrad M. Freuling, Andreas Hlinak, Christoph Schulze, Julia Sehl-Ewert, Patrick Wysocki, Claudia A. Szentiks, Klaus Schmitt, Peter Wohlsein, Gesa Kluth, Ilka Reinhardt, Thomas C. Mettenleiter, Thomas Müller

**Affiliations:** 1 Institute of Molecular Virology and Cell Biology, Friedrich-Loeffler-Institut, 17493 Greifswald- Insel Riems, Germany; 2 Berlin-Brandenburg State Laboratory, 15236 Frankfurt (Oder), Germany; 3 Department of Experimental Animal Facilities and Biorisk Management, Friedrich-Loeffler-Institut, 17493 Greifswald- Insel Riems, Germany; 4 Friedrich-Loeffler-Institut, Institute of Epidemiology, 17493 Greifswald- Insel Riems, Germany; 5 IZW - Leibniz Institute for Zoo and Wildlife Research, 10315 Berlin, Germany; 6 Landesamt für Verbraucherschutz Saarland, 66115 Saarbrücken, Germany; 7 Department of Pathology, University of Veterinary Medicine Hannover, Foundation, 30559 Hannover, Germany; 8 LUPUS – German Institute for Wolf Monitoring and Research, 02826 Görlitz, Germany; 9 Friedrich-Loeffler-Institut, 17493 Greifswald- Insel Riems, Germany

**Keywords:** Pseudorabies, Aujeszky’s disease, Suid herpesvirus, Wild boars, Dogs, Foxes, Wolves

## Abstract

**Background:**

The high susceptibility of carnivores to Suid Alphaherpesvirus 1 [SuAHV1, synonymous pseudorabies virus (PrV)], renders them inadvertent sentinels for the possible occurrence of Aujeszky’s disease (AD) in domestic and wild swine populations. The aim of this study was to epidemiologically analyse the occurrence of PrV infections in domestic and wild animals in Germany during the last three decades and to genetically characterise the causative PrV isolates.

**Methods:**

PrV in dogs was detected using standard virological techniques including conventional and real time PCR, virus isolation or by immunohistochemistry. Available PrV isolates were characterized by partial sequencing of the open gC reading frame and the genetic traits were compared with those of archived PrV isolates from carnivores and domestic pigs from Germany before the elimination of AD in the domestic pig population.

**Results:**

During 1995 and 2022, a total of 38 cases of AD in carnivores, e.g. dogs and red foxes, were laboratory confirmed. Sequencing and subsequent phylogenetic analysis of PrV isolates established a strong connection between AD cases in carnivores and the occurrence of PrV infections in European wild boars in the end phase of and after elimination of AD from the domestic pig population. While PrV infections occur at low numbers but regularly in hunting dogs, interestingly, PrV was not observed in grey wolves in Germany. In none of 682 dead-found grey wolves and wolf-dog hybrids tested from Germany during 2006–2022 could PrV infection be detected by molecular means.

**Conclusions:**

Although PrV has been eliminated from domestic pigs, spillover infections in domestic and wild carnivores should always be expected given the endemic presence of PrV in wild pig populations. Since detection of PrV DNA and virus in carnivores is sporadic even in areas with high seroprevalence of PrV in wild pigs, it may not reflect the full diversity of PrV.

## Introduction

Pseudorabies virus (PrV), an enveloped double-stranded DNA virus and member of the genus *Varicellovirus* within the subfamily *Alphaherpesvirinae* of the *Orthoherpesviridae* family [1], is the causative agent of Aujeszky’s disease (AD), an infection of major economic impact in animal husbandry [2]. Although its taxonomic name Suid alphaherpesvirus 1 (SuAHV1) clearly indicates its natural association with swine, the virus exhibits a wide host range capable of infecting basically all mammals except higher primates and equines [2]. While only swine are able to survive a productive infection and are thus considered the only natural host, virus infections in other mammalian species, i.e. non-natural hosts, are fatal due to virus neuroinvasion and lethal inflammatory response [3, 4]. In general, such spillovers represent dead-end infections as the non-reservoir hosts are not able to independently maintain infection because of their rapid fatal outcome [5, 6].

AD has been eradicated from populations of domestic swine through use of culling and/or vaccination programs in many countries in Europe, in North America, Australia and New Zealand [2, 7–9] but still remains a serious problem in other parts of the world. However, PrV continues to circulate among free-roaming or farmed wild boar and feral swine, which can act as a reservoir for the virus [10–13]. PrV infections in populations of wild boar have been confirmed for several European countries [11, 13] but also a few countries in Northern Africa [14], and Asia [15–18]. PrV is also present in populations of feral swine in the United States [12] and Brazil [19]. There is evidence that PrV isolates of wild boar and feral swine origin in Europe and the US do not represent a homogenous population but rather represent several different genetic lineages [7, 20]. The geographical distribution of PrV in wild swine is rather patchy including both large-scale transboundary but also small cluster occurrence. Depending on the region, PrV seroprevalences in wild boar and feral swine can amount to 50% or even higher [11–13].

Because subclinical infections and nonspecific clinical signs are common in swine, the high susceptibility of carnivores renders them inadvertent sentinels for virus occurrence. Often detection of PrV in dogs (*Canis lupus familaris*) may be the first indication that the virus is present in a swine herd or a local wild swine population [21]. Dogs that live on pig farms may become infected after direct or indirect contact with infected swine, while hunting dogs are especially prone to infection by direct exposure to feral swine during hunting events. Consumption of uncooked offals from infected (wild) swine also plays a role as a source of infection [22–24]. Once infected, dogs die 6 to 96 h after the onset of clinical neurological signs [25].

Although PrV spillover infections are observed most commonly in farm dogs, in recent years cases of PrV in hunting dogs after direct contact with wild boars have been repeatedly reported from Europe [20, 26–33], the United States [34–36] and China [37]. PrV infections have also been reported in wildlife [38] including farmed and free-living foxes (*Vulpes vulpes*) [39–42], endangered carnivores such as the Florida panther (*Puma concolor couguar*) [43], grey wolf (*Canis lupus*) [44–46], Iberian lynx (*Lynx pardinus*) [47], African wild dogs (*Lycaon pictus*) [48] and captive brown bears (*Ursus arctos*) [49] after consumption of PrV-contaminated meat.

In this study, we describe occurrence of PrV infections in domestic dogs and wild carnivores in Germany during the past three decades, with a subsequent effort to genetically characterize isolates based on partial sequence analyses. Furthermore, we wanted to elucidate whether PrV infections in wild boar pose a threat to the rising, highly protected population of grey wolves in Germany.

## Materials and methods

### Epidemiological information and sampling

Because AD is a notifiable infectious disease in Germany [9], any suspect cases in animals have to be submitted to regional veterinary laboratories for laboratory diagnosis. The number of laboratories confirmed PrV cases in carnivores for the period 1995–2022 was obtained from the electronic Animal Disease Reporting System (TSN) of the competent veterinary authorities of the districts and federal states and the Federal Ministry of Food and Agriculture [50]. PrV isolates or viral DNA of PrV PCR-positive dogs and wild carnivores were submitted by regional veterinary laboratories to the national reference laboratory (NRL) for AD at the FLI for confirmation and further molecular characterization.

As part of a research project of the Leibnitz Institute for Zoo and Wildlife Research (IZW), Berlin, on the causes of mortality in grey wolves, brain samples from wolves and wolf-dog hybrids in Germany from 2006 to 2022 were submitted to the NRL at the Friedrich-Loeffler-Institut (FLI) and tested for PrV. Master data for wild carnivore samples such as date and geographical origin (Gauss-Krueger coordinates) were obtained from TSN or the IZW and DBBW (federal documentation and advisory centre on wolves) database (https://www.dbb-wolf.de/). Georeferencing of data and map visualization was done using the ArcGIS 10.8 package.

### PRV diagnostics

Standard virological techniques were used to detect PrV infection [51]. Viral DNA in brain samples and/or cell culture supernatant was detected following DNA extraction using the MagNA Pure LC Total Nucleic Acid Isolation Kit for automated extraction (Roche Diagnostics Deutschland GmbH, Mannheim, Germany) or DNA-MiniKit (Qiagen, Hilden, Germany) by both conventional PCR [52, 53] and triplex real time PCR either using the gB- and gE-gene (variant 1) specific or the UL19 (major capsid protein gene)- and gE-gene (variant 2) specific assay including respective internal control DNA (IC2) essentially as described [54]. Isolation of PrV from PCR positive brain tissues was conducted in cell cultures. To this end, brain samples were homogenized using a TissueLyser (Qiagen, Hilden, Germany), resuspended in Dulbecco’s minimum essential medium (DMEM), zentrifuged at 1000 g and the supernatant incubated at 37 °C on different cell lines. Cell lines generally used for PrV isolation at regional veterinary laboratories included rabbit kidney (RK-13), porcine kidney (PK-15), Madin–Darby bovine kidney (MDBK), bovine oesophagus (KOP), embryonic porcine kidney epithelial (SPEV) or primate Vero cells as established. Cells were observed for a minimum of seven days and passaged three times to confirm a negative result [55].

When appropriate, PrV in brain tissue was detected by immunohistochemistry (IHC) using a polyclonal PrV rabbit hyper immune serum (Vemie, Kempen, Germany) or an in-house polyclonal rabbit antibody against PrV gB [56]. Positive antigen detection was visualized via avidin-biotin-peroxidase-complex (ABC; Vector Laboratories, Burlingame, USA) with 3,3´-Diaminobenzidintetrahydrochlorid (DAB) or 3-amino-9-ethylcarbazole (AEC) as chromogen, respectively [56, 57]. Tissue sections were further stained with hematoxylin-eosin (HE) to evaluate PrV-induced lesions.

### Sequencing and alignment

For the characterisation of genetic traits and for phylogenetic analysis, archived PrV isolates from carnivores and domestic pigs from Germany before the elimination of AD in 2003 were also included.

Sequencing PrV isolates or DNA was done essentially as described [20]. Briefly, DNA was prepared using commercial DNA extraction kits (Qiagen, Germany) from cell culture or brain tissue. After amplification of a 732 bp fragment comprising parts of the gC open reading frame using using pfx DNA polymerase (Invitrogen, Germany). PCR products were separated on 1% agarose gels, purified using the Genomic DNA Purification Kit (Thermo Fisher Scientific, Germany) and sequenced on both strands using the Big Dye R terminator cycle sequencing kit (Applied Biosystems, Darmstadt, Germany) with the primers used for amplification. Sequences were aligned and their evolutionary history was inferred using the Neighbor-Joining method [58] as implemented in MEGA X [59]. Nucleotide sequences generated from hunting dogs and red foxes in this study were submitted to GenBank. Accession numbers are shown in Table 1. For comparison, additional PrV sequences of domestic pigs and wild boars from previous studies were included in the phylogenetic analysis.

**Table 1:** Details to PrV isolates used for sequencing and subsequent phylogenetic analysis

Isolate	Year	Country	Host	Accession/ Submission-ID	Reference
GER 34 BW	1983	Germany	Felis catus (domestic cat)	2706745	This study
GER 57 ST	1992	Germany	Canis lupus familiaris (domestic dog)		This study
GER 7 BRB	1994	Germany	Sus scrofa familiaris (domestic pig)		This study
GER 25 BRB	1994	Germany	Canis lupus familiaris (domestic dog)		This study
GER 40 NI	1994	Germany	Canis lupus familiaris (domestic dog)		This study
GER 7 BRB	1994	Germany	Canis lupus familiaris (domestic dog)		This study
GER 12 BRB	1995	Germany	Sus scrofa (wild boar)	GQ259094.1	[19]
GER 13 BRB	1995	Germany	Sus scrofa (wild boar)	GQ259095.1	[19]
GER 11 ST	1996	Germany	Sus scrofa (wild boar)	GQ259093.1	[19]
GER 15 BRB	1996	Germany	Sus scrofa (wild boar)	GQ259096.1	[19]
GER 615 SN	2009	Germany	Canis lupus familiaris (domestic dog)	2706745	This study
GER 618 MWP	2010	Germany	Canis lupus familiaris (domestic dog)		This study
GER 619 SN	2010	Germany	Canis lupus familiaris (domestic dog)		This study
GER 550 NRW	1999	Germany	Sus scrofa (wild boar)	GQ259102.1	[19]
GER 551 NRW	1999	Germany	Sus scrofa (wild boar)	GQ259103.1	[19]
GER 552 NRW	1999	Germany	Sus scrofa (wild boar)	GQ259104.1	[19]
GER 553 RP	2000	Germany	Canis lupus familiaris (domestic dog)	GQ259105.1	[19]
GER 555 RP	2000	Germany	Sus scrofa (wild boar)	GQ259107.1	[19]
GER 556 NRW	2000	Germany	Sus scrofa (wild boar)	GQ259108.1	[19]
GER 554 RP	2002	Germany	Canis lupus familiaris (domestic dog)	GQ259106.1	[19]
GER 611 RP	2003	Germany	Canis lupus familiaris (domestic dog)	GQ259116.1	[19]
GER 613 SN	2005	Germany	Sus scrofa (wild boar)	GQ259118.1	[19]
GER 614 BW	2008	Germany	Canis lupus familiaris (domestic dog)	GQ862778.1	[19]
AUT 620*	2011	Austria	Canis lupus familiaris (domestic dog)	2706745	This study
GER 622 LS	2011	Germany	Canis lupus familiaris (domestic dog)		This study
GER 626 RP	2015	Germany	Canis lupus familiaris (domestic dog)		This study
GER 632 RP	2017	Germany	Canis lupus familiaris (domestic dog)		This study
GER 634 SR	2017	Germany	Canis lupus familiaris (domestic dog)		This study
GER 635 SR	2017	Germany	Canis lupus familiaris (domestic dog)		This study
GER 636 LS	2017	Germany	Canis lupus familiaris (domestic dog)		This study
GER 637 TH	2017	Germany	Canis lupus familiaris (domestic dog)		This study
GER 638 RP	2018	Germany	Canis lupus familiaris (domestic dog)		This study
GER 641 SR	2019	Germany	Vulpes vulpes (red fox)		This study
GER 642 BRB	2019	Germany	Vulpes vulpes (red fox)		This study

*dog tested PrV positive in Germany, but was infected during hunting activities in Austria.

## Results

### PrV cases in carnivores

**Fig. 1:**
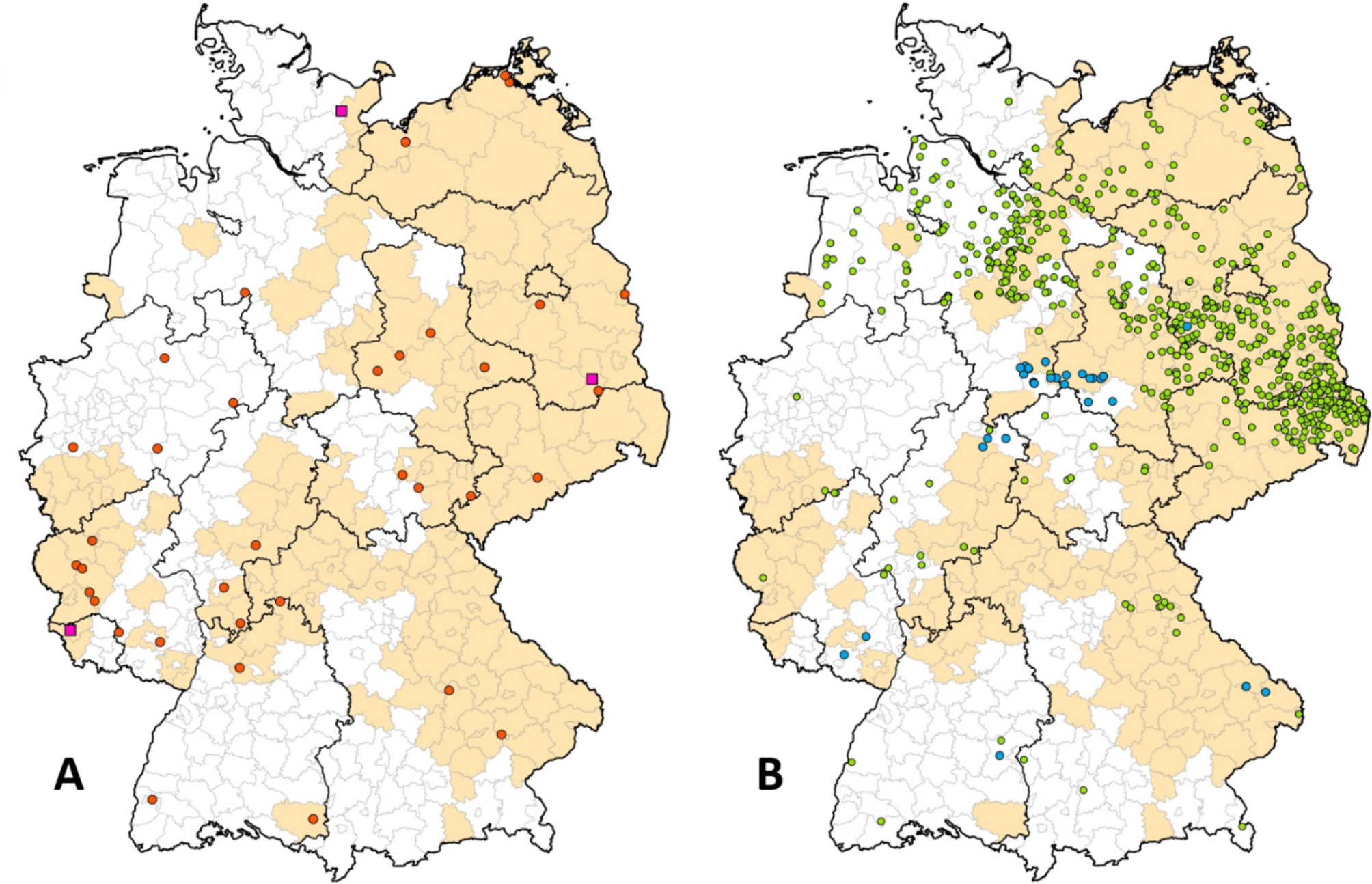
**A**: Distribution of reported PrV-infections in dogs (dots) and foxes (rectangles) in Germany between 1995 and 2022. **B**: Locations of grey wolves and lynxes submitted for PrV diagnosis between 2006 and 2022. The borders of the federal states are indicated. Areas where PrV is endemic in wild boar are coloured beige and are based on a recent publication [57]

**Fig. 2:**
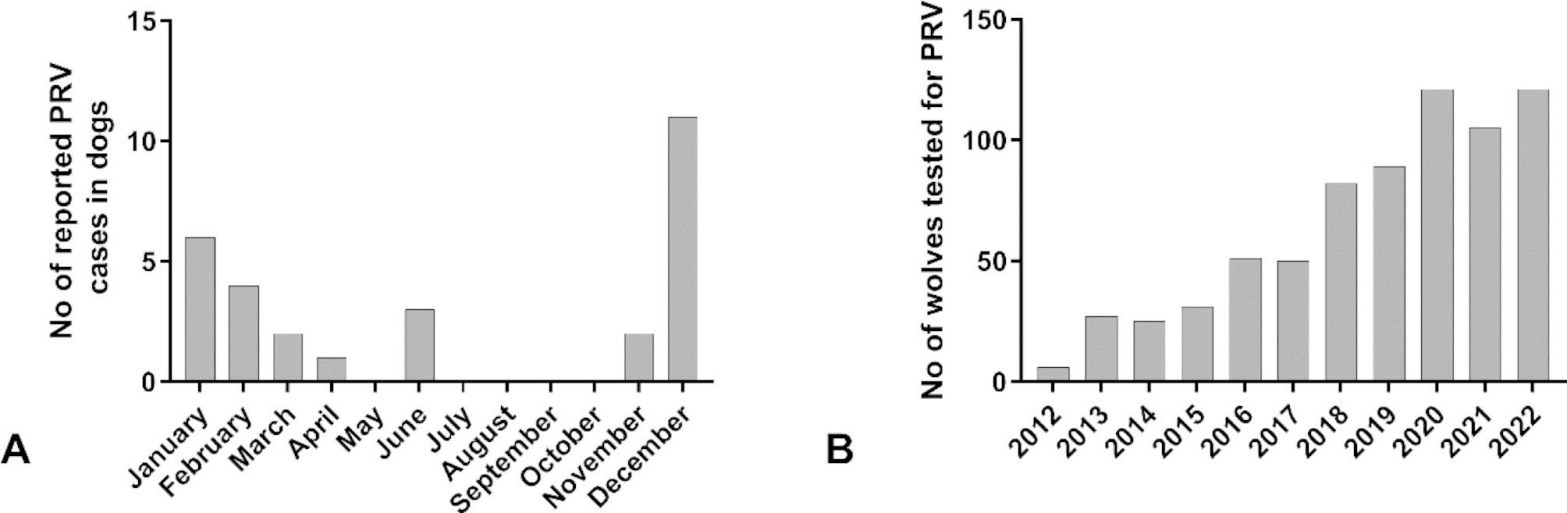
**A**: Monthly distribution of PrV cases in dogs for the time period 1995–2022. **B**: Number of wolfes investigated between 2006 and 2022

Between 1995 and 2022, PrV infections were reported in a total of 35 dogs and three foxes in Germany (Fig. 1A), with higher incidences in December and March (Fig. 2A). While the affected dogs showed an acute course of disease associated with severe neurological signs including pruritus and self-mutilation, PrV in foxes was detected during routine rabies surveillance as a differential diagnosis. Foxes originated from the federal states of Saarland, Schleswig-Holstein and Brandenburg (Fig. 1A). Twenty-seven (77.1%) and 2 (5.7%) of the 35 PrV cases in dogs could be epidemiologically linked to direct contact with wildlife and consumption of uncooked offal, respectively, while for the remaining dogs the source of infection was unclear.

In the frame of the research project on causes of mortality in grey wolves in Germany, during the period 2006–2022 at total of 682 grey wolves and wolf-dog hybrids, 31 lynxes (*Lynx lynx*) (Figs. 1B and 2B), 24 red foxes (*Vulpes vulpes*), three golden jackals (*Canis aureus*), 15 stone and pine martens (*Martes foina, martes*), four European polecats (*Mustela putorius*), four raccoons (*Procyon lotor*), and three badgers (*Meles meles*) were tested. In none of the animals could PrV be detected.

### Clinical and pathomorphological findings

**Fig. 3:**
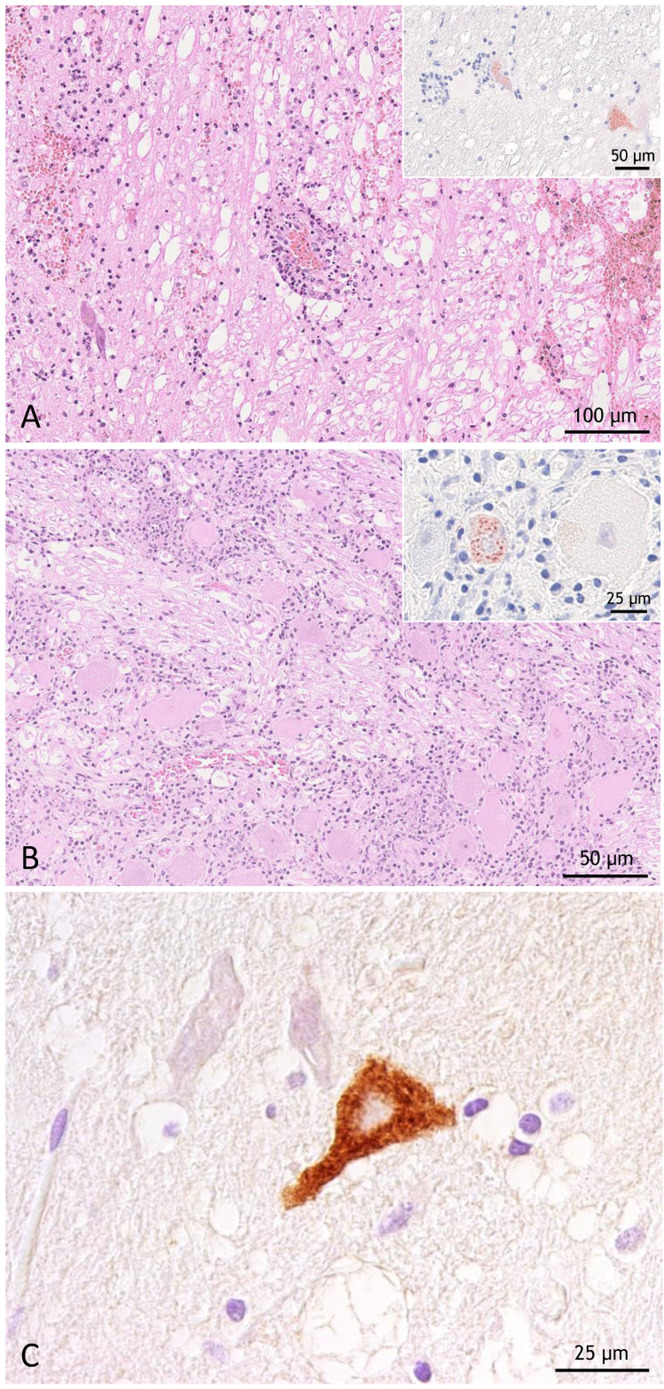
Histopathology of PrV-infected dogs (**A** and **B**) and a red fox (**C**). (**A**) Brainstem encephalitis with vasculitis, perivasculitis, hemorrhage and scattered PrV antigen positive neurons (inset), HE and anti-PrV gB immunohistochemistry [53]. (**B**) Trigeminal ganglionitis with an immunopositive neuron (inset), HE and anti-PrV gB immunohistochemistry. (**C**) Brain showing a PrV infected neuron in a red fox (GER 642), immunohistochemistry [54].

Two of 35 PrV positive dogs were investigated clinically and pathomorphologically. Dog 1 presented with central nervous signs including tremor, paralysis and pruritus, hypothermia and hypersalivation. Clinical and pathological investigation revealed delayed coagulation, hemolysis, hematuria and electrolyte imbalances. The dog died shortly (4 days) after onset of clinical signs. On necropsy, the dog showed hemoperitoneum, hemothorax, black-coloured stomach content, gastric mucosal hemorrhages, melaena as well as hemorrhages of the mediastinum and mesenterium. Histopathologically, moderate mixed-cellular encephalitis with vasculitis and perivasculitis, gliosis, scattered neuronal necrosis and extensive hemorrhages was found in the brainstem. Mononuclear infiltrates, neuronal necrosis and hemorrhages were also detected in the trigeminal ganglion. Immunopositive neurons and glial cells were detected within and adjacent to the affected areas (Fig. 3A). Dog 2 presented with pruritus, licking, central nervous signs, hypersalivation and somnolence leading to euthanasia. Gross pathology revealed reddened skin with dermatitis of a paw and pulmonary alveolar edema. Histopathologically, inflammation was limited to the trigeminal ganglion which showed mild mixed-cellular infiltration and occasional neuronal necrosis. Positive signals for viral antigen were only rarely detected in the trigeminal ganglion, but were more frequently found in brainstem neurons (Fig. 3B).

One of the three PrV positive foxes from Brandenburg was found dead by a hunter in November 2017 and submitted for testing on notifiable and reportable infections including rabies, canine distemper and PrV. At necropsy, no lesions except for a few wild boar bristles in the stomach were detected suggesting acute death due to circulatory breakdown. Bacteriological investigations as well as virological and parasitological screening for rabies, canine distemper, canine adenovirus (CadV-1, CadV-2), *Toxoplasma gondii*, and leptospirosis yielded negative results, while the fox was infested with *Toxocara canis* and *Echinococcus multilocularis*. PrV infection was confirmed by realtime PCR and virus isolation. While histological investigations revealed no inflammatory reactions, very few PrV infected neurons were detected by subsequent IHC staining (Fig. 3C).

The PrV positive red fox from Saarland was found in January 2019 in an agonal state showing extreme pruritus, and was humanely euthanized in a local veterinary clinic. While rabies could be excluded by routine diagnostics, PrV was confirmed by standard virological methods. For the remaining PrV positive fox from Schleswig-Holstein no further information on the history and possible source of infection was available.

### Phylogenetic analysis

**Fig. 4:**
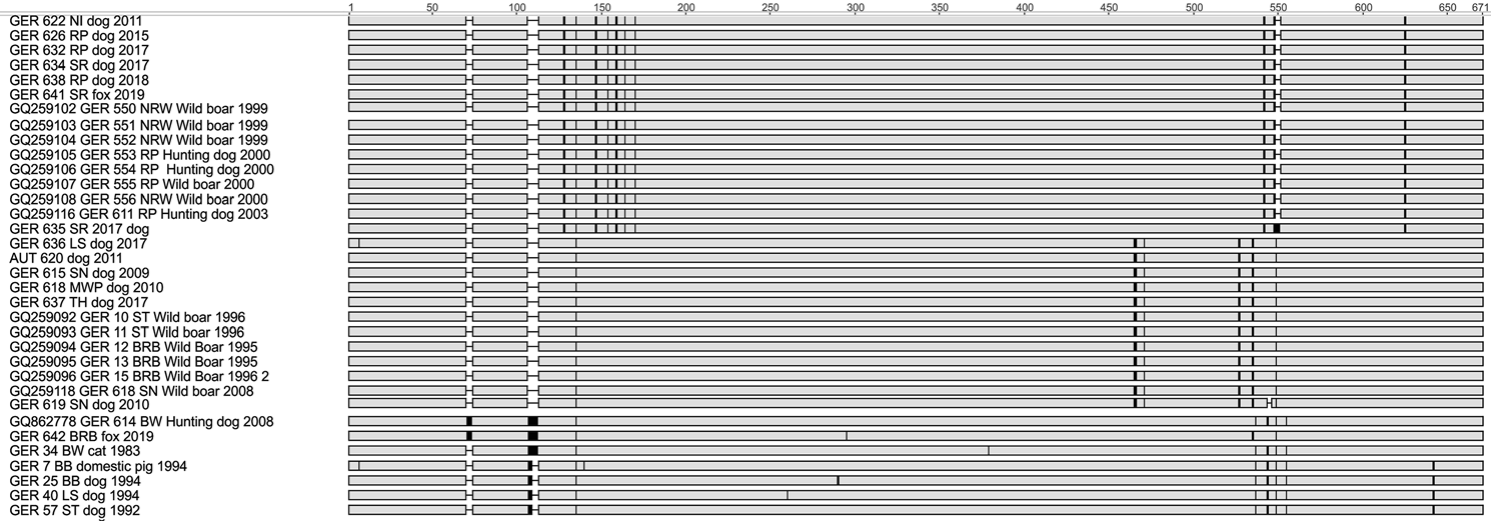
Symbolized alignment of the partial gC-sequence from German PrV-isolates, with identical nucleotides displayed as gray bar and SNPs and indels indicated (black)

Sequence analysis of PrV isolates from Germany identified different variants according to a limited number of sequence variations, i.e. SNPs and deletions and insertions (Fig. 4).

**Fig. 5:**
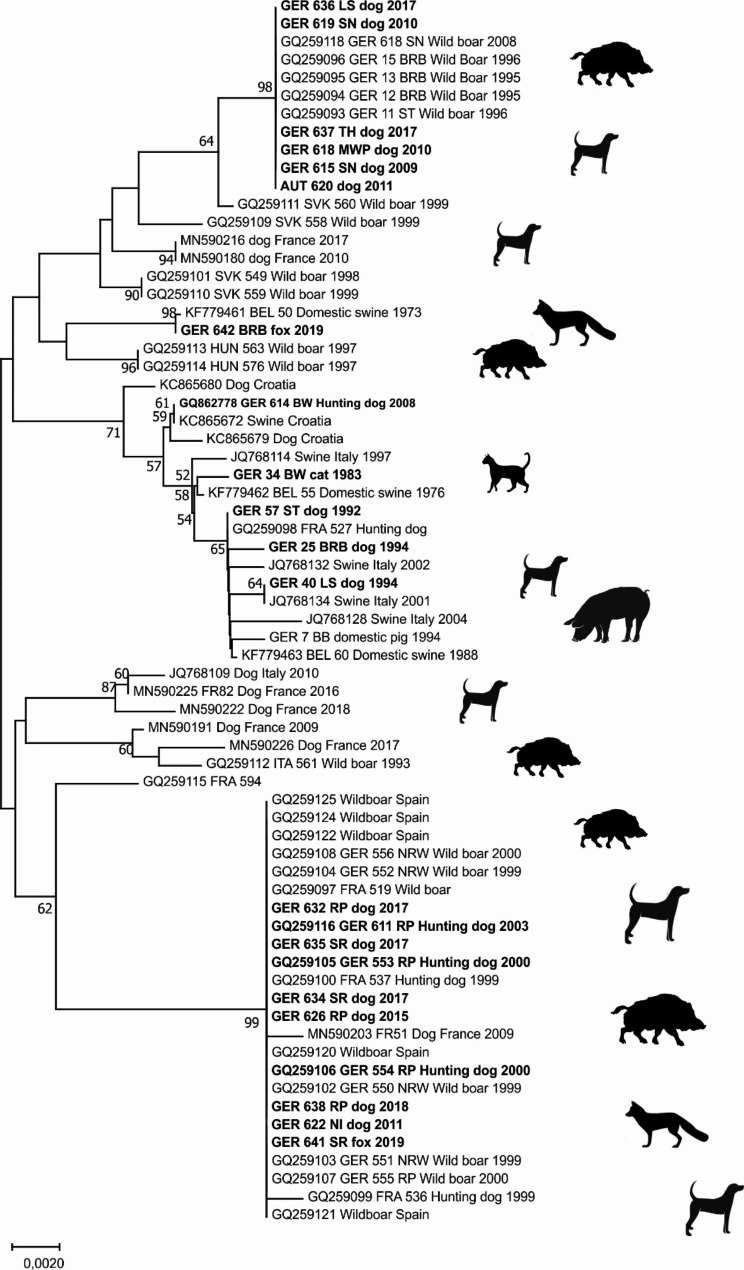
Unrooted Neighbour-Joining tree based on (gaps removed) from the PrV-gC coding region. Sequences are identified by: GenBank accession number/strain/country/species/year of isolation (if known). German PrV strains from carnivores are in highlighted (bold). Numbers along the branches represent percentages of 500 bootstrap iterations with values over 50% shown.

Phylogenetic analyses revealed that AD in carnivores in Germany was caused by different PrV strains. Carnivore PrV isolates originating from prior to the elimination of AD from domestic pig populations in Germany group together with PrV from pigs (Fig. 5). More recent PrV isolates from carnivores largely clustered closely with the prevailing PrV strains found in European wild boars. A number of PrV from dogs and one fox from Saarland are within the previously assigned Clade “B” comprising of isolates from Germany (North-Rhine Westphalia, Rhineland-Palatinate), France and Spain. The only exception is a hunting dog from Baden-Württemberg (GER 614), which has an identical sequence to a PrV from a domestic pig from Croatia (GenBank: KC865672). PrV from dogs from the eastern German federal states are identical to the identified variant from the European wild boar, which was designated as part of lineage “A”. Interestingly, a fox isolate from Brandenburg (GER 642) clusters with a domestic pig PrV isolate from Belgium. The sequence of this isolate shares two indels with a hunting dog from Baden-Wurttemberg (GER 614), but also incorporates unique SNPs (Fig. 4).

## Discussion

Although AD has been eliminated from the domestic pig population in Germany [9], PrV infections are enzootic in European wild boar populations throughout the country [60–63] causing an estimated overall PrV seroprevalence of 12.09% (Fig. 1) [62]. Our study confirms similar studies from other European countries [20, 26–32, 64] with a sporadic number of reported PrV infections in domestic carnivores (Figs. 1A and 2 A). Occasionally, PrV from wild boars caused cases in dogs, mainly hunting dogs, during the past decades as reported in the Animal Disease Reporting System (TSN), with a temporal link to hunting activities. This association was confirmed by partial sequence analyses using the gC-gene (Figs. 4 and 5) that had been used to characterize wild boar derived PrV isolates from Europe [20]. In contrast, historic cases of PrV in cats and dogs prior to the elimination of AD from domestic pigs that were also included in this study, were caused by the prevailing domestic pig PrV lineages at the time (Fig. 5).

Wild boar associated PrV cases in dogs are more likely after direct contact during hunting than oral ingestion of offal. Likely, increased stress levels in latently infected wild boar may cause reactivation of virus replication and active shedding without eliciting clinical signs [65], eventually leading to an infection in hunting dogs when they actively encounter wild boar during hunting activities [11]. Up to 7% of wild swine in endemic areas of Florida were PCR positive in nasal, oral and genital swabs indicating low levels of PrV shedding [66]. Oro-nasal infection with direct brain manifestation likely only requires a low infection dose, but also ingestion of wild boar offal led to a clinical PrV infection in captive wolves [46].

Against this background it is interesting that despite other reports none of the 682 investigated free-ranging wolves and hybrids submitted between 2006 and 2022 tested positive for PrV (Fig. 2B), despite the overlap of their distribution in Germany in areas with high PrV seroprevalences in wild boars, high wild boar densities [67, 68] and their natural behavior to prey on wild boars [69, 70] (Fig. 1B). Certainly, one limitation is that testing on PrV only relied on brain tissues but did not include peripheral nerve system (PNS) samples, e.g. trigeminal ganglia (TG) and dorsal root ganglia (DRG). Based on the pathogenesis of PrV-induced neuropathic itch, PrV remains in the PNS and the infection will cause a systemic inflammatory response that will lead to the death of non-natural hosts. Hence, brain samples will not be systematically positive for PrV [4, 38].

Studies of scat samples from Europe, on the other hand, indicate a certain flexibility of the wolf as a predator depending of the availability of prey, which significantly increases the chances of becoming infected with PrV, as is the case with hunting dogs. While in northern plain lands of Europe wolves seem to rather avoid wild boar as prey [71], in the Mediterranean basin wild boars are obviously sometimes part of the prey [69, 70]. In a recent study, diet of wolves from Italy was consistently dominated by the consumption of wild boar which accounted for about two-thirds of total prey biomass [72]. Data from Germany show that about 20% of total prey biomass of wolves consists of wild boar [73]. According to official statistics, the main causes of death of grey wolves found dead between 2006 and 2022 are traffic accidents (74.8%), followed by illegal killing (9.1%), natural death (8.8%) and management (authorised removal of animals after incidents, 1.2%). In 5.5% and 1.2% of the cases, the cause of death is unclear and still under investigation, respectively (https://www.dbb-wolf.de/totfunde/statistik-der-todesursachen). Since infection of grey wolves with PrV would inevitably lead to a clinically visible manifestation and eventually death, the absence of PrV-infected grey wolves in our sample suggests that PrV infections are very rare in wolves and the few occasional infections may have gone undetected.

In contrast, we report three cases of PrV in free-ranging red foxes. In one case, the fox was shot by a hunter as it showed atypical behavior suggestive of encephalitis, which was confirmed by immunohistopathology (Fig. 3C), while in other studies from Germany investigating hunted foxes, no PrV infections were detected [74, 75]. Since red foxes are known to feed on carcasses as well as to prey on European wild boar piglets [76], the oral route of infection is the most plausible.

While the virus found in one red fox (GER 641) from the western federal state of Saarland was identical in its partial DNA sequence to other PrV isolates from the previously established “clade B” [20], isolate GER 642 from eastern Brandenburg clusters with a dog from Baden-Württemberg and is identical in its partial gC-gene sequence with a Belgian domestic pig isolate from 1973 (Fig. 5). This finding indicates/emphasizes that the phylogenetic clustering pattern needs to be interpreted with caution [29]. This is not only based on low bootstrap support, as indicated before [29], but also on the apparent stability of the gC-gene with limited sites of genetic diversity (Fig. 4). Whether other PrV genes are better suited for phylogenetic analysis remains to be demonstrated, if even whole-genome sequences do not provide a better resolution [77].

Furthermore, sampling biases and surveillance gaps may suggest epidemiological links where in reality there are none. As regards isolate GER 642, this red fox isolate serves as an indicator for the presence of a yet unknown PrV variant in this part of Germany bordering Poland, a country where no information on PrV characterization in wildlife is available. These results are similar to findings in Austria where the genetic diversity of PrV was only evident after investigating PrV from hunting dogs [29]. Alternative hypotheses, e.g. that the fox was PrV infected in areas endemic with a different virus, are not plausible given the short incubation period and the average home range of red foxes. Also, since Germany is free of AD [9], consumption of infected offal from domestic pigs is extremely unlikely. Against the background of the limited usefulness of gC-gene-based phylogeny for epidemiological inference, PrV cases in red foxes that had been described before [40, 41] may need reconsideration.

## Conclusion

With their prominent clinical picture, PrV infections in carnivores are likely to be discovered and virus can be characterized, as seen in this study and others [27, 29, 31]. In contrast, even in areas with high seroprevalence of PrV in wild boar, the detection of PrV DNA and virus in carnivores is sporadic and may not disclose the full diversity of circulating PrV. Therefore, both brain and PNS samples from carnivores with suspected encephalitis should be thoroughly investigated [4], to exclude infection with rabies virus (RABV) but also to analyze for PrV, even though the infection is only regulated in domestic pigs in the EU (EU regulation (EU) 2018/1882).

## Data Availability

All data generated or analysed during this study are included in this published article [and its supplementary information files].
